# The hydrogen-bond network of water supports propagating optical phonon-like modes

**DOI:** 10.1038/ncomms10193

**Published:** 2016-01-04

**Authors:** Daniel C. Elton, Marivi Fernández-Serra

**Affiliations:** 1 Department of Physics and Astronomy, Stony Brook University, Stony Brook, New York 11794-3800, USA; 2 Institute for Advanced Computational Science, Stony Brook University, Stony Brook, New York 11794-3800, USA

## Abstract

The local structure of liquid water as a function of temperature is a source of intense research. This structure is intimately linked to the dynamics of water molecules, which can be measured using Raman and infrared spectroscopies. The assignment of spectral peaks depends on whether they are collective modes or single-molecule motions. Vibrational modes in liquids are usually considered to be associated to the motions of single molecules or small clusters. Using molecular dynamics simulations, here we find dispersive optical phonon-like modes in the librational and OH-stretching bands. We argue that on subpicosecond time scales these modes propagate through water's hydrogen-bond network over distances of up to 2 nm. In the long wavelength limit these optical modes exhibit longitudinal–transverse splitting, indicating the presence of coherent long-range dipole–dipole interactions, as in ice. Our results indicate the dynamics of liquid water have more similarities to ice than previously thought.

## Abstract (web summary)

How the local structure of water varies as a function of temperature is a long-studied topic, which is still under debate. Here, the authors show that dielectric susceptibility measurements might be used to probe and identify propagating optical phonon-like modes in the hydrogen-bond network of water.

## Graphical abstract

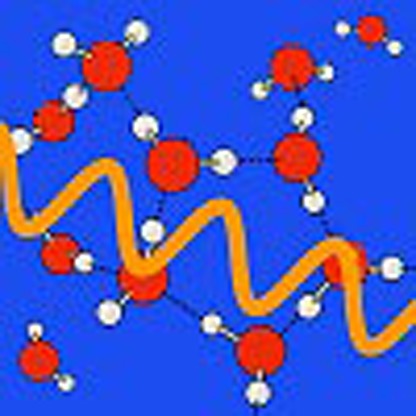

The local structure and dynamics of liquid water as a function of temperature remains a source of intense research and lively debate^1^,^2^,^3^,^4^,^5^,^6^. A thus far unrecognized discrepancy exsits between the peak assignments reported in Raman spectra with those reported in dielectric/infrared spectra. Although early experimentalists fit the Raman librational band with two peaks^7^, it is better fit with three (Supplementary Table 1)^8^,^9^,^10^,^11^,^12^. Previously these three peaks were assigned to the three librational motions of the water molecule—twisting (≈435 cm^−1^), rocking (≈600 cm^−1^) and wagging (≈770 cm^−1^)^8^,^9^,^11^. However, when comparing these assignments with infrared and dielectric spectra, one runs into a serious discrepancy. One expects to find the two higher frequency modes to be present, since only the rocking and wagging librations are infrared active. The twisting libration, consisting of a rotation of the hydrogen atoms around the C2 axis, is not infrared active since it does not affect the dipole moment of the molecule. Instead, infrared spectra show two peaks at 380 and 665 cm^−1^ (ref. 13), and similarly dielectric spectra show peaks at 420 and 620 cm^−1^ (ref. 14), in disagreement with this assignment.

The assignment of longitudinal optical phonon modes to Raman spectra can be made by looking at the longitudinal dielectric susceptibility. This method has been used previously to assign longitudinal phonon modes to the Raman spectra of ice Ih^15^,^16^,^17^, cubic ice^18^ and vitreous GeO_2_ and SiO_2_ (ref. 19). It has previously been shown that the librational peak in the longitudinal dielectric susceptibility of water is dispersive^20^, and Bopp and Kornyshev noted that the dispersion relation has the appearance of an optical phonon mode^21^. The longitudinal mode in the dielectric susceptibility is equivalent to the dispersive mode discovered by Ricci *et al.*^22^ in the spectrum of hydrogen-density fluctuations^22^.

Comparison of peak positions in longitudinal and transverse dielectric susceptibilities often reveals longitudinal–transverse (LO–TO) splitting. LO–TO splitting indicates the presence of long-range dipole–dipole interactions in the system. One way to understand LO–TO splitting is through the Lyddane–Sachs–Teller (LST) relation^23^:


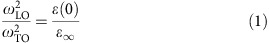


Although this relation was originally derived for a cubic ionic crystal it was later shown to have very general applicability^24^,^25^, and has been applied to disordered and glassy solids^17^,^26^,^27^. To apply this equation to water we must use a generalized LST relation, which takes into account all of the optically active modes in the system and the effects of dampening^24^. The generalized LST relation is^24^:


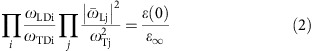


Here the index *i* runs over the Debye peaks in the system and the index *j* runs over the number of damped harmonic oscillator peaks. The longitudinal frequencies of the damped harmonic oscillators must be considered as complex numbers 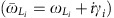, where *γ*_*i*_ is the dampening factor.

As shown by Barker, the generalized LST equation can be understood purely from a macroscopic point of view^24^, so by itself it yields little insight into microscopic dynamics. LO–TO splitting can be understood from a microscopic standpoint via the equation^28^,^29^:


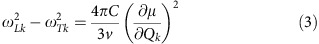


Here *v* is the volume per unit cell, *Q*_*k*_ is the normal coordinate of mode *k*, and *C* is a prefactor which depends on the type of lattice and the boundary conditions of the region being considered (for an infinite cubic lattice, *C*=1). Equation 3 shows that LO–TO splitting is intimately related to crystal structure, and it has been used to evaluate the quasi-symmetry of room temperature ionic liquids^30^.

In this work we show how the dielectric susceptibility can be used to probe water's local structure and dynamics. Our work solves the aforementioned peak assignment discrepancy. We find that the lowest frequency librational Raman peak (≈435 cm^−1^) is a transverse optical phonon-like mode while the highest frequency peak (≈770 cm^−1^) is a longitudinal optical phonon-like mode. This explains why the highest frequency Raman mode does not appear in infrared or dielectric experiments, since such experiments only report the transverse response. We show that the transverse counterpart also exhibits dispersion. We argue that these dispersive modes are due to optical phonons that travel along the H-bond network of water. Our results indicate that not only does water exhibit LO–TO splitting, but also that its dependence with temperature is anomalous. We suggest that this measurement provides an alternative probe to evaluate structural changes in liquid water as a function of temperature.

## Results

### LO–TO splitting from experimental data

As in our previous work^31^ we compared results from a rigid (TIP4P/*ɛ*) model, a flexible model (TIP4P/2005f), and a flexible and polarizable model (TTM3F) in all of our analyses.

We wish to study the *k* dependence of the dielectric susceptibility, where *k*=2*π*/*λ*. *k*− dependence cannot be probed directly by experiment, but in the limit of infinite wavelength (*k*→0) the longitudinal and transverse dielectric susceptibilities can be obtained from the dielectric function via the following relations^32^,^33^:


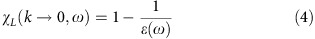



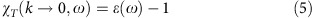


Note that the transverse susceptibility is what one normally calls susceptibility. The dielectric function can be obtained from the index of refraction *n*(*ω*) and extinction coefficient *k*(*ω*) as:


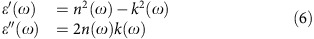


**Figure 1:**
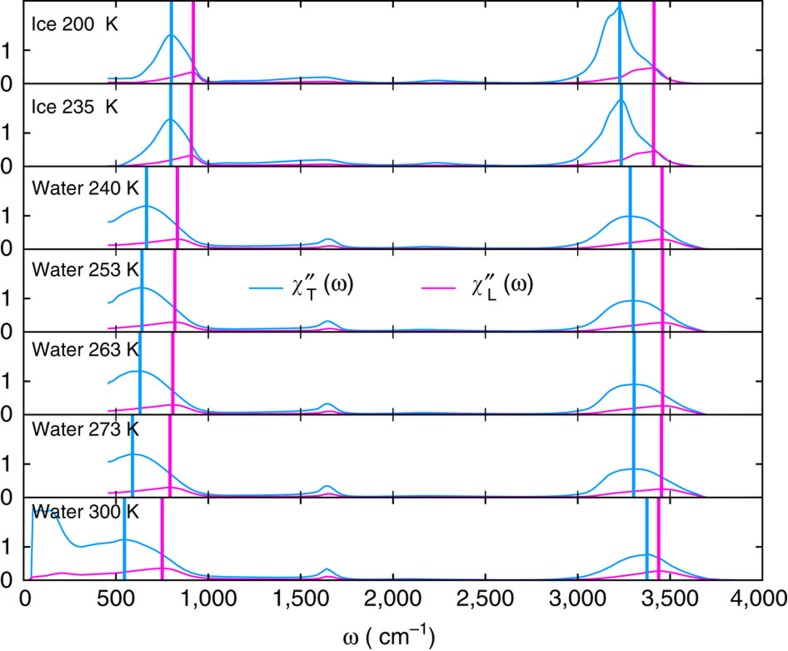
Dielectric susceptibilities of ice and water. Computed from index of refraction data using equations 4 and 6. data from 210 to 280 K comes from aerosol droplets^34^ while the data at 300 comes from bulk liquid^35^.

These equations allow us to use previously published experimental data^34^,^35^ to calculate the imaginary part of the longitudinal response. We find significant LO–TO splitting in the librational and stretching bands (Fig. 1).

### Polarization correlation functions

The normalized longitudinal and transverse polarization correlation functions are defined as:


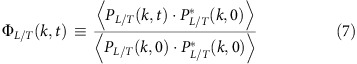


**Figure 2:**
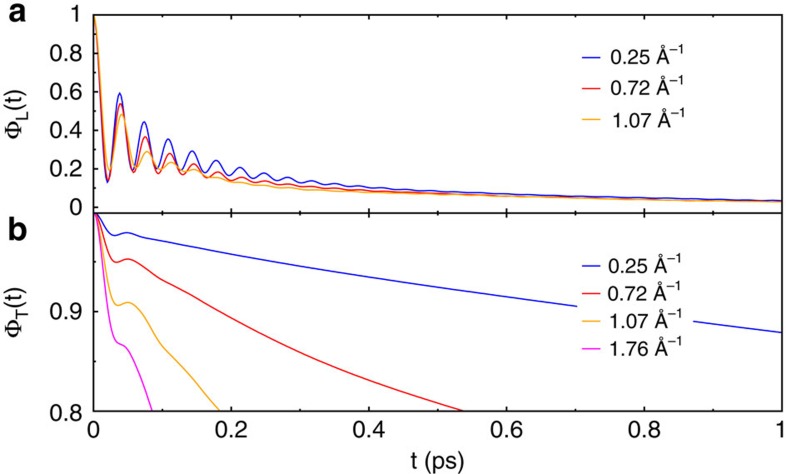
Polarization correlation functions. Longitudinal (**a**) and transverse (**b**) polarization correlation functions (see equation 7) for TIP4P/*ɛ*, a rigid model. The oscillations at small *k* come from the collective librational mode, which is much more pronounced in the longitudinal case.

The correlation functions found for TIP4P/*ɛ* at small small *k* are shown in Fig. 2. Since TIP4P/*ɛ* is a rigid model, only librational motions are present. The addition of flexibility and polarizability add additional high-frequency oscillations to the picture (Supplementary Fig. 1). In the small wavenumber regime (*k*<1.75 Å) there is a damped oscillation, which corresponds to the collective librational phonon-like mode. This damped oscillation is superimposed on an underlying exponential relaxation in both the transverse and longitudinal cases. In the longitudinal case the relaxation time *τ*(*k*) of the underlying exponential relaxation exhibits non-monotonic behaviour with *k*, reaching a maximum at *k*≈3 Å^−1^ (Supplementary Fig. 2). At wavenumbers greater than *k*≈2.5 Å only intramolecular motions contribute.

### Dispersion of the librational peak

**Figure 3:**
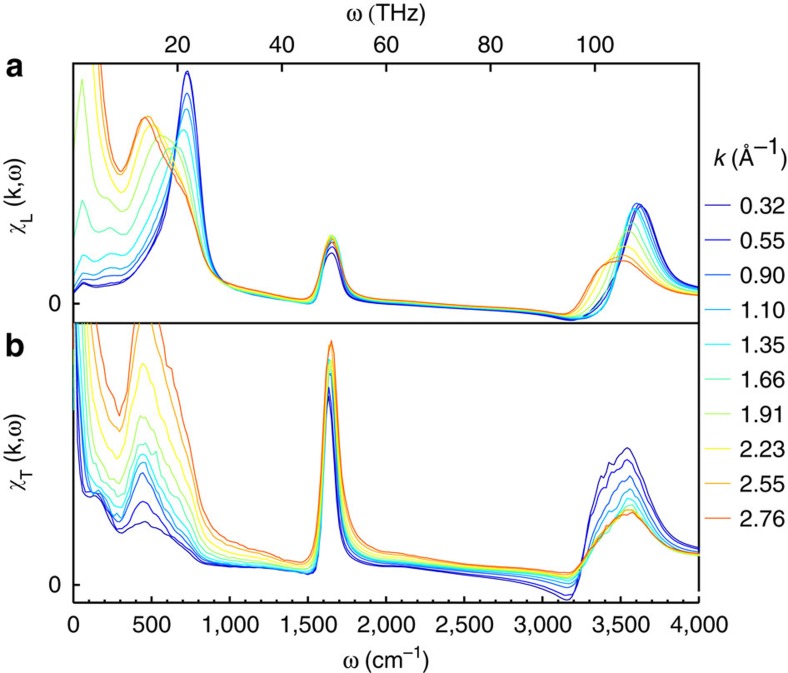
Imaginary part of longitudinal and transverse susceptibility. (**a**) Transverse and (**b**) longitudinal susceptibility from simulations with TTM3F at 300 K. In the longitudinal spectra both the librational (∼750 cm^−1^) and OH-stretching peak (∼3,500 cm^−1^) peaks exhibit dispersion.

**Figure 4:**
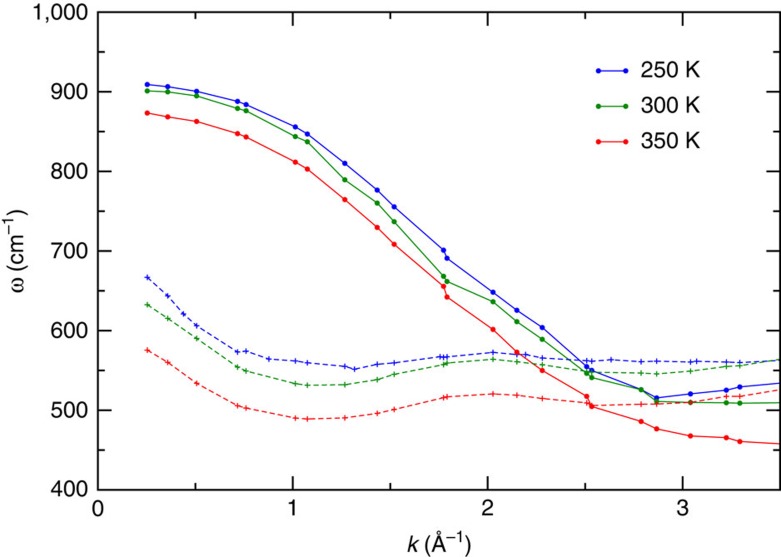
Dispersion relations for the propagating librational modes. For TIP4P/2005f at three different temperatures (squares=longutudinal, pluses=transverse). A similar plot was found for TTM3F, but with lower frequencies.

Figure 3 shows the imaginary part of the longitudinal and transverse susceptibility for TTM3F. In the longitudinal case the librational peak is clearly seen to shift with *k*. In the transverse case, the lower frequency portion of the band is seen to shift slightly with *k*. Dispersion relations for the longitudinal and transverse librational peaks are shown in Fig. 4 for three different temperatures, using one peak fits. The dispersion relations appear to be that of optical phonons. In both the longitudinal and transverse case the dampening factors remain less than the resonance frequencies, indicating an underdamped oscillation (Supplementary Fig. 3). The longitudinal dispersion relation for TIP4P/2005f agrees with that found by Bopp and Kornyshev (who used the flexible BJH model)^21^. Resat *et al.*^36^ also obtained a similar dispersion relation (but at a higher frequency), using the reference memory function approximation for TIP4P instead of molecular dynamics.

Resonance frequencies and lifetimes for the smallest *k* are shown in Table 1. The speed of propagation of these modes was computed by finding the slope d*ω*/d*k* in the regime of linear dispersion. For TIP4P/2005f we found speeds of ≈2,700 and ≈1,800 m s^−1^ for the longitudinal and transverse modes. These propagation speeds are above the speed of sound in water (1,500 m s^−1^) but below the speed of sound in ice (4,000 m s^−1^). The temperature dependence of the propagation speed was found to be very small.

In both the longitudinal and transverse cases, the residual of the peak fitting shows features not captured by our Debye+one resonance fit of the librational peak. In both the longitudinal and transverse cases there is a non-dispersive peak at higher frequency, located at ≈900 cm^−1^ for TIP4P/2005f and at ≈650 cm^−1^ in TTM3F. This peak is negligibly small in the *k*=0 longitudinal susceptibility but appears as a shoulder as *k* increases. In the transverse case the overlapping peak persists at *k*=0, so we found that the *k*=0 transverse spectra is best fit with two peaks, in agreement with experimental spectra. As we describe later, the higher frequency transverse peak is largely due to the self part of the response and is associated with the wagging librations of single molecules.

### Importance of polarizability

**Figure 5:**
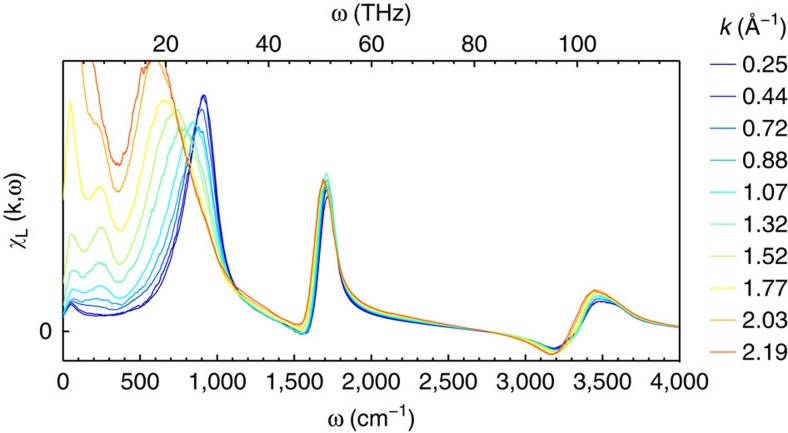
Imaginary part of the longitudinal susceptibility. For TIP4P/2005f at 300 K. No significant dispersion is observed in the OH-stretching peak.

**Figure 6:**
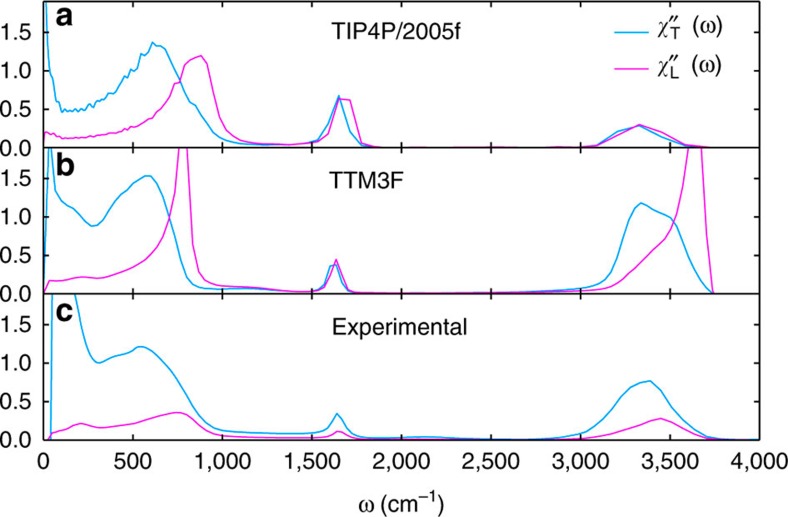
Imaginary parts of dielectric susceptibility. We compare (**a**) the non-polarizable model TIP4P/2005f, (**b**) the polarizable model TTM3F, and (**c**) experimental data^35^ at 298 K. The effects of polarization can be seen in the LO–TO splitting of the stretching mode and in the low-frequency features.

There are several notable differences between TTM3F and the non-polarizable model TIP4P/2005f. First of all, the librational band of TIP4P/2005f is at higher frequency, in worse agreement with experiment. This difference in frequency is likely related to the parameters of TIP4P/2005f and not its lack of polarization. More importantly, we find that TTM3F exhibits dispersion in the OH-stretching band (≈3,500 cm^−1^) in the longitudinal case, while TIP4P/2005f does not (Fig. 5). The transverse susceptibility of TTM3F does not exhibit such dispersion but the magnitude of the OH-stretching band increases at small *k*, indicating long-range intermolecular correlations. TIP4P/2005f does not exhibit this behaviour. Similarly, at *k*=0 TTM3F exhibits significant LO–TO splitting in the OH-stretching band, while TIP4P/2005f does not (Fig. 6). These findings are consistent with Heyden *et al.*'s results for the *k*-resolved infrared spectra from *ab initio* simulation, where they concluded that polarization allows for intermolecular correlations at the OH-stretch frequency^37^.

These findings can be understood from the dipole derivative in equation 3. In the librational band the derivative of the dipole moment with respect to normal coordinate is purely due to rotation, while in the OH-stretching band it is due to changes in the geometry of the molecule and electronic polarization of the molecule during the OH-stretching. In principle, there may be coupling between the librational and stretching motions, but typically such rotational–vibrational coupling effects are negligibly small^38^. The dipole moment surface (fluctuating charges) and polarization dipole incorporated in TTM3F account for the changes in polarization that occur during OH-stretching motion. These results confirm the significance of polarization in capturing the OH-stretching response of water^37^.

Figure 6 shows a comparison of TTM3F, TIP4P/2005f and experiment at *k*=0. While the location of the peaks in TTM3F are in good agreement with the experimental data at 298 K, the magnitude of the longitudinal response is greatly overestimated in TTM3F. The degree of LO–TO splitting in the OH-stretching peak is also overestimated in TTM3F. In general it appears that TTM3F overestimates the dipole derivative in equation 3, while TIP4P/2005f underestimates it. Figure 6 also shows the effect of polarization at low frequencies, in particular the appearance of an H-bond stretching response at ≈250 cm^−1^ in TTM3F, which is absent in TIP4P/2005f (ref. 31).

### LO–TO splitting versus temperature

The frequencies of the librational and stretching modes are shown in Table 1. Once again we compare our results to experimental data^34^,^35^,^39^. The comparison is imperfect since the TIP4P/2005f and TTM3F data come from data at finite *k* (the smallest *k* in the system). For all three systems (TIP4P/2005f, TTM3F and experiment) the increase in the LO–TO splitting of the librational band is puzzling, since the right hand side of the LST relation predicts a decrease in splitting, corresponding to a smaller dielectric constant and weaker dipole–dipole interactions. We found verifying the generalized LST equation is difficult because water contains either two or three Debye relaxations which must be taken into account^40^,^41^. Uncertainties in how to fit the region of 1–300 cm^−1^ (0.2–9 THz), which includes contributions from many H-bonding modes, precludes a direct application of the generalized LST relation to water. By ignoring this region, however, we were able to achieve an approximate validation of the generalized LST equation for TIP4P/2005f. A more detailed analysis of how to fit the low-frequency region will be the focus of future work. Since the generalized LST equation is an exact sum rule it can be used to assist in testing the validity of different fit functions.

### Relation to phonons in ice

Naturally we would like to find corresponding optical phonon modes in ice. As shown in Fig. 1 the dielectric spectra and LO–TO splitting of supercooled water resembles that of ice. Recently evidence has been presented for propagating librational phonon modes in ice XI (refs 42, 43). Three of the twelve librational modes of ice XI are infrared active (labelled WR1, RW1 and RW2) and all three exhibit LO–TO splitting. The splittings have been found from Raman scattering to be 255, 135 and 35 cm^−1^ (ref. 43). These modes all consist of coupled wagging and rocking motions. The WR1 mode, which has the largest infrared intensity, most closely matches our results. WR1 and RW2 have the same transverse frequency and RW1 has a smaller infrared intensity, which may help explain why the librational band is well fit by a single optical mode. LO–TO splitting in the OH-stretching modes of hexagonal ice has been discussed previously^17^.

### Range of propagation

The range of propagation of these modes can be calculated as *R*=*τv*_*g*_ where *τ* is the lifetime and *v*_*g*_=d*ω*/d*k* is the group velocity. For TIP4P/2005f we find a range of propagation of ≈1.1 nm for the longitudinal librational mode and ≈0.3 nm for the transverse mode. Similar results hold for TTM3F.

To verify that the modes we observe are actually propagating and to further quantify the range of propagation we study the spatial extent of polarization dipole correlations as a function of frequency. We investigated several different methodologies that can be used to decompose a spectra into distance-dependent components (Supplementary Note 1). We choose to start with the polarization correlation function:


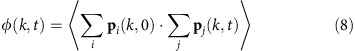


Here **p**_*i*_(*k*, *t*) represents either the longitudinal or transverse molecular polarization vector of molecule *i*. We now limit the molecules in the second sum to those in a sphere of radius *R* around each molecule *i*:


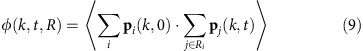


The resulting function exhibits the expected *R*→0 limit, yielding only the self-contribution. *R* can be increased to the largest *R* in the system 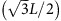, where the full response function for the simulation box is recovered. As *R* increases, the contributions of the distinct term add constructively and destructively to the self term, illustrating the contributions from molecules at different distances.

**Figure 7:**
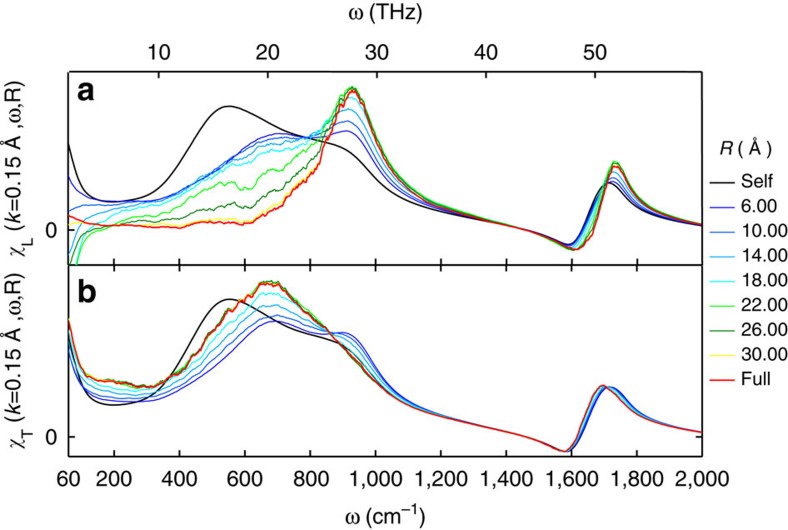
Imaginary part of the distance decomposed susceptibility for TIP4P/2005f. Transverse (**a**) and longitudinal (**b**) susceptibilities, calculated with a 4 nm box at 300 K, using the smallest **k** vector in the system. Gaussian smoothing was applied. Long-range contributions to the librational peak extending to *R*=2 nm are observed.

**Figure 8:**
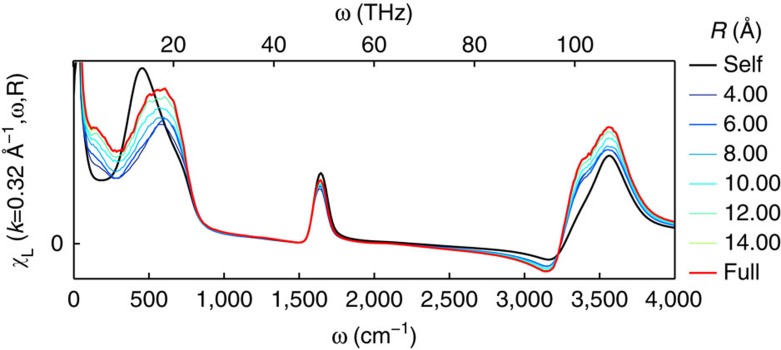
Imaginary part of the distance decomposed longitudinal susceptibility for TTM3F at 300 K. Long-range contributions are observed in the OH-stretching band.

Figure 7 shows the distance decomposed longitudinal and transverse susceptibilities for TIP4P/2005f in a 4-nm box at the smallest *k* available in the system. Similar results are obtained for TTM3F, but with additional contributions in the stretching band. Figure 8 shows the distance decomposed longitudinal susceptibility for TTM3F in a 1.97 nm box. The entire region between 0–1,000 cm^−1^ contains significant cancellation between the self and distinct parts, in qualitative agreement with a previous study^44^ (Fig. 8). In the longitudinal susceptibility, the self component has two peaks (at 500 and 900 cm^−1^ for TIP4P/2005f) representing the two infrared active librational motions (rocking and wagging, respectively). The self part is the same in both the longitudinal and transverse cases, reflecting an underlying isotropy, which is only broken when dipole–dipole correlations are introduced. Further insight into the self-distinct cancellation comes from the results of Bopp, *et al.*, who project the hydrogen currents into a local molecular frame, allowing them to study the cross correlations between the rocking and wagging librations^21^. They find that in the longitudinal case cross correlations between rocking and wagging contribute negatively in the region of 480 cm^−1^ and positively in the region of 740 cm^−1^, suppressing the lower frequency peak to zero and enhancing the higher frequency peak.

In both the transverse and longitudinal cases as *R* increases a new peak emerges, corresponding to the propagating mode. Incidentally, the shift in the peak between the self and distinct parts rules out the possibility that the propagating mode is the proposed dipolar plasmon resonance, since the dipolar plasmon must be a resonance of both the of single molecule and collective motion^45^,^46^,^47^. Interestingly, there are very long-range contributions to this peak. In our simulations with a 4-nm box of TIP4P/2005f contributions persist up to 3 nm in the longitudinal case and 2 nm in the transverse case. As noted, recent studies of ice XI suggest that the propagating modes consist of coupled wagging and rocking librations^42^,^43^. The results for the transverse mode seem to confirm this hypothesis for liquid water, since the propagating mode peak lies between the single-molecule rocking and wagging peaks. In the longitudinal case the propagating mode overlaps more with the wagging peak, suggesting a greater role for these type of librations in the longitudinal phonon.

### Methanol and acentonitrile

To provide further evidence the aforementioned optical modes propagate through the hydrogen-bond network of water we decided to repeat our analysis for other polar liquids, both H-bonding and non H-bonding. As an H-bonding liquid we choose methanol, which is known to contain winding H-bonded chains. According to results from MD simulation, most of these chains have around 5–6 molecules^48^,^49^, with a small percentage of chains containing 10–20 molecules^50^. Chain lifetimes have been estimated to be about 0.5 ps (ref. 50). Therefore, we expect methanol can also support a librational phonon mode that propagates along hydrogen bonds, but perhaps with a shorter lifetime and range than water. As a non H-bonding polar liquid we choose acetonitrile, because it has a structure similar to methanol, but with the hydroxyl group replaced by a carbon atom. We find that the OH librational band of methanol (≈700 cm^−1^ (ref. 51)) is indeed dispersive (Supplementary Fig. 4). As with water, the transverse spectra also exhibits dispersion, but to a much lesser extent. LO–TO splitting of about 100 cm^−1^ is observed in the 700 cm^−1^ librational peak. The results for acetonitrile are more ambiguous—we observe dispersion in the broad peak at ≈100 cm^−1^, however, this peak contains contributions from translational and (free) rotational modes, as well as the CH_3_ torsion mode, and it is not clear, which modes are responsible for the dispersion (Supplementary Fig. 5).

## Discussion

In this work, we have presented several lines of evidence for short-lived optical phonons that propagate along the H-bond network of water. The longitudinal and transverse nonlocal susceptibility exhibit dispersive peaks with dispersion relations resembling optical phonons. As the temperature is lowered, the resonance frequencies and LO–TO splittings of these modes converge towards the values for phonons in ice Ih. By comparing our results with a recent study of ice XI we believe both modes likely consist of coupled wagging and rocking librations^42^,^43^.

This work fundamentally changes our understanding of the librational band in the Raman spectra of water by assigning the lower and higher frequency peaks to transverse and longitudinal optical modes. Our analysis of the self-distinct cancellation indicates that the middle Raman peak (≈600 cm^−1^) belongs to the remnant of the single-molecule wagging response, which remains after the cancellation. We are also led to a new interpretation the librational region of the real part of the dielectric function. In the case of a lossless optical phonon the transverse phonon occurs where *ɛ*′(*ω*)=∞, while the longitudinal phonon occurs where *ɛ*′(*ω*)=0. The presence of dampening smooths the divergence leading to a peak followed by a sharp dip. This is what is observed in the real part of the dielectric function of water between 300 and 500 cm^−1^ (the features are shifted to lower frequencies by the tail of the low-frequency Debye relaxation).

One might wonder how our work relates to existing work on acoustic modes in water, in particular, the controversial ‘fast sound' mode^52^,^53^. Acoustic modes, which are observable through the dynamic structure factor, have been explored as means of understanding the hydrogen-bond structure and low-temperature anomalies of water^5^. In this work, we have argued that optical modes can also provide insight into water's structure and dynamics. The fast sound mode lies at much lower frequencies than the librational and OH stretch modes that we studied. The H-bond bending and stretching modes also primarily lie at at frequencies below the librational region. However, normal mode analysis of liquid water and clusters shows that the H-bond stretching modes have a wide distribution of frequencies, which overlaps with the librational modes, so some coupling between these modes is possible^54^,^55^. Recently, it was shown that there is coupling between the acoustic and optic modes in water—that is, between fluctuations in mass density and fluctuations in charge density^56^.

The large spatial range and coherent propagation of these modes is surprising and implies the existence of an extended hydrogen-bond network, in contrast to earlier ideas about the structure of water which emphasize dynamics as being confined within small clusters^57^. Simulations with larger simulation boxes are needed to fully quantify the extent of the longitudinal modes. The ability of water to transmit phonon modes may be relevant to biophysics, where such modes could lead to dynamical coupling between biomolecules, a phenomena that is currently only being considered at much lower frequencies^58^,^59^,^60^. The methodology used in this paper to analyse LO–TO splitting opens up a new avenue to understanding the structure and dynamics of water. The fact that the librational LO–TO splitting increases with temperature instead of the expected decrease is likely due to significant changes in the structure of the liquid. One likely possibility is that the volume per ‘unit cell' term in equation 3 decreases with temperature. This could be caused by the local quasi-structure determined by H-bonding changing from a more ice-like structure (four molecules per unit cell) to a more cubic structure (1 molecule per unit cell). More research is needed to understand the microscopic origin of the LO–TO splitting in water, both in the librational and stretching modes.

## Methods

### Theory of the nonlocal susceptibility

If the external field is sufficiently small, then the relation between the polarization response of a medium and the electric displacement field *D* for a spatially homogeneous system is given by:


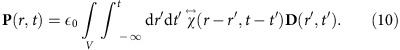


This expression Fourier transforms to:


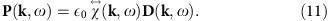


For isotropic systems, the tensor 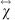 can be decomposed into longitudinal and transverse components:


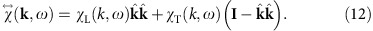


The easiest starting point for deriving microscopic expressions for *χ*_*L*_(*k*, *ω*) and *χ*_*T*_(*k*, *ω*) is the classical Kubo formula^61^:


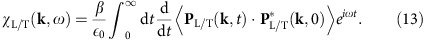


This expression relates the susceptibility to the time correlation function of the polarization in equilibrium. The longitudinal part of the polarization can be calculated by Fourier transforming the defining expression for the polarization ∇̇**P**(**r**, *t*)=−*ρ*(*r*,*t*), leading to 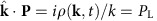. To calculate the transverse part of the polarization we use the method of Raineri and Friedman to find the polarization vector of each molecule (Supplementary Note 2)^62^. We can rewrite equation 13 in terms of the normalized polarization correlation function (equation 7), and taking into account the isotropy of water:


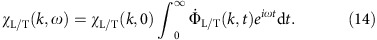


### Computational methods

The three water models we used were TIP4P/*ɛ* (ref. 63), TIP4P/2005f (ref. 64) and TTM3F (ref. 65). To simulate methanol and acetonitrile we used the General AMBER Forcefield (GAFF)^66^, a forcefield with full intramolecular flexibility, which has been shown to satisfactory reproduce key properties of both liquids^67^. Our TTM3F simulations were performed with an in-house code that uses the TTM3F force calculation routine of Fanourgakis and Xantheas. All other simulations were ran using the GROMACS package (ver. 4.6.5)^68^. We used particle–mesh Ewald summation for the long-range electrostatics with a Coloumb cutoff of 2 nm for our 4+nm simulations and a cutoff of 1.2 nm for our simulations with 512 molecules. Our TTM3F simulations had 256 molecules and used Ewald summation with a Coulomb cutoff of 0.9 nm. The principle TIP4P/2005f simulations contained 512 molecules and were 8 ns long (Δ*t*_out_=8 fs) and 0.6–1.2 ns long (Δ*t*_out_=4 fs). Other simulations were 1–2 ns long. Simulations with MeOH and ACN contained 1,000 molecules and were 1-ns long. All simulations were equilibrated for at least 50 ps before outputting the data.

Because of periodic boundary conditions, the possible **k** vectors are limited to the form 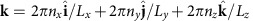, where *n*_*x*_, *n*_*y*_ and *n*_*z*_ are integers. We calculated correlation functions separately for each **k** and then average over the results for **k** vectors with the same magnitude, a process we found reduced random noise.

One can question whether a purely classical treatment is justified here because the librational dynamics we are interested have frequencies of 700–900 cm^−1^ for which *ℏω*≈3–4*k*_*B*_*T* at 300 K. Previously it was shown that the widely used harmonic correction does not change the spectrum^21^. Furthermore, comparison of *k*-resolved infrared spectra taken from molecular dynamics and *ab inito* density functional theory (DFT) simulation show that they give qualitatively similar results for all frequencies below 800 cm^−1^ (ref. 37). For the OH-stretching peak, however, quantum effects are known to be very important.

### Fitting the librational band

To obtain resonance frequencies and lifetimes for the librational peak in the imaginary part of the response we used a damped oscillator model. A Debye peak overlaps significantly with the librational band in both the longitudinal and transverse cases and must be included in the peak fitting. Equation 14 can be used to relate the form of the time correlation function to the absorption peak lineshape. For Debye response one has the following expressions:


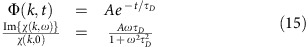


For resonant response with resonance frequency *ω*_0_(*k*) and dampening factor *γ*≡1/*τ* we have:


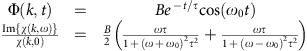


We find this lineshape (the Van Vleck–Weisskopf lineshape^69^,^70^) yields results identical to the standard damped harmonic oscillator response for the range of *τ*,*ω*_0_ values we are interested in. We found a two function (Debye+resonant) fit worked very well for fitting the librational peak in the longitudinal case (Supplementary Figs 6 and 7). The H-bond stretching peak at ≈200 cm^−1^ overlaps with the librational band for 2<*k*<2.5, and we found that it can be included in the fit using an additional damped harmonic oscillator, but usually this was not necessary. Because of this overlap and due to the broad nature of the transverse band, the fitting in the transverse case is only approximate. We found this was especially true for TTM3F and the experimental data, so we do not report lifetimes for such cases.

## Additional information

**How to cite this article:** Elton, D. C. & Fernández-Serra, M. The hydrogen-bond network of water supports propagating optical phonon-like modes. *Nat. Commun.* 7:10193 doi: 10.1038/ncomms10193 (2016).

## Supplementary Material

Supplementary Information Supplementary Figures 1-7, Supplementary Table 1, Supplementary Notes 1-2 and Supplementary References

## Figures and Tables

**Table 1:** Resonance frequencies and lifetimes.

**Model**	**Temp**	***ω***_***LO***_	***τ***_***LO***_	***ω***_***TO***_	***τ***_***TO***_	***ω***_**LO**_**−*****ω***_**TO**_
TIP4P/2005f	250	905	0.38	667	0.23	233
	300	900	0.44	632	0.18	268
	350	871	0.34	574	0.18	297
	400	826	0.25	423	0.17	400
3*TTM3F	250	757	0.49	496		261
	300	721	0.44	410		311
	350	710	0.20	380		330
expt^34^	253	820		641		179
expt^35^	300	759		556		203

Frequencies are given in cm^−1^ and lifetimes in ps. The values from simulation were computed at the smallest *k* in the system. The experimental values are based on the position of the max of the band and therefore only approximate.
