# Supplementary material for: The hydrogen-bond network of water supports propagating optical phonon-like modes
Source: Nat Commun. 2016 Jan 4;7:10193. doi: 10.1038/ncomms10193 (PMC4725765; doi:10.1038/ncomms10193)
Supplement: Supplementary Information — Supplementary Figures 1-7, Supplementary Table 1, Supplementary Notes 1-2 and Supplementary References [file ncomms10193-s1.pdf]

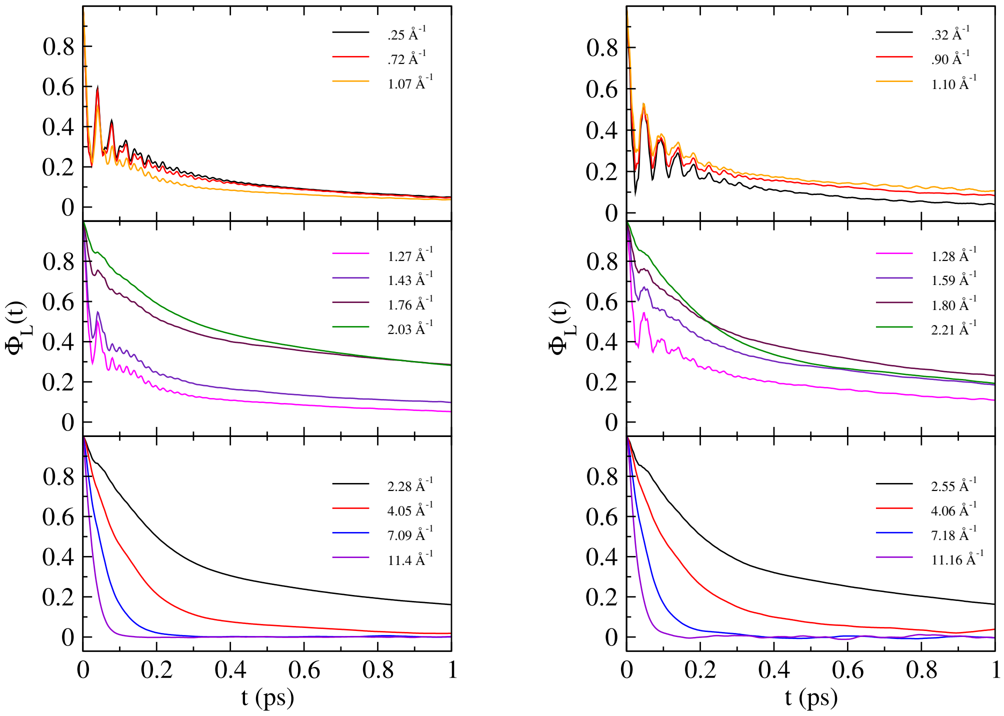

Supplementary Figure 1: **Longitudinal polarization relaxation functions.** Shown for 512 TIP4P2005/f (left) and TTM3F (right) at 300 K.

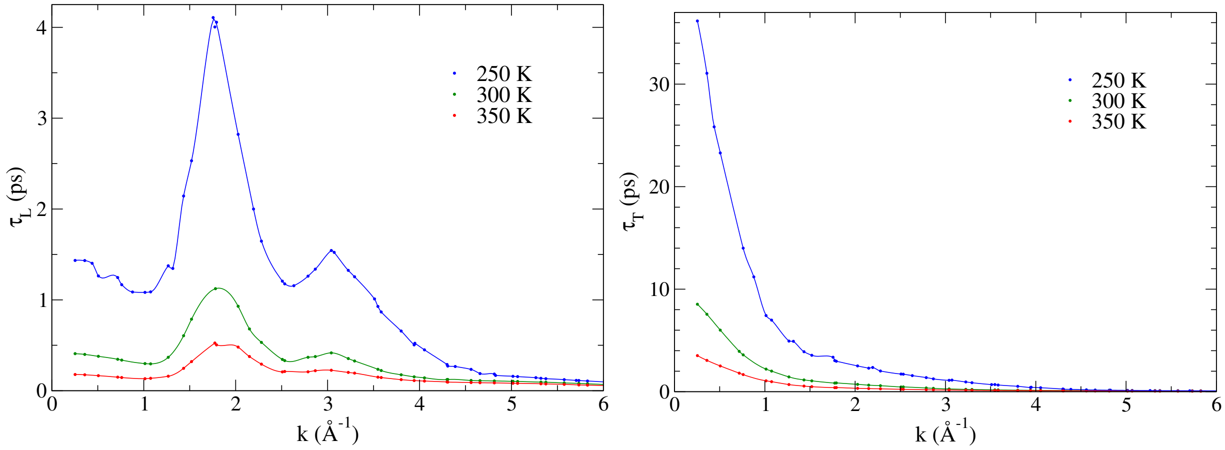

Supplementary Figure 2: **Longitudinal (left) and transverse (right) relaxation times for 512 TIP4P/2005f.** Computed for the underlying exponential of the relaxation. The points are interpolated by Akima splines. The transverse relaxation time here is equal to the Debye relaxation time, which at $k = 0$ is ≈ 11 ps at 300 K for TIP4P/2005f. Experimentally it is 8.5 ps.[1]

A. Dispersion relations and dampening factors

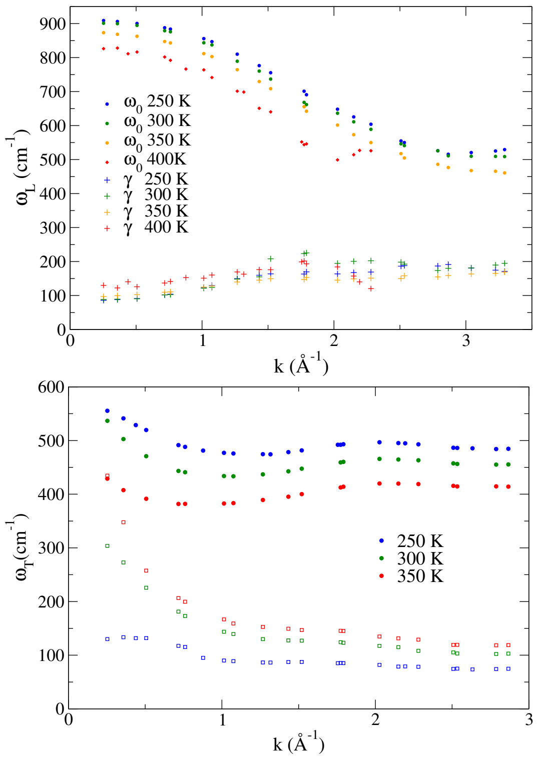

Supplementary Figure 3: **Longitudinal (top) and transverse (bottom) dispersion relations (circles) and dampening factors (squares) for 512 TIP4P/2005f.** These curves were obtained from a two peak (Debye + resonant) fit. In contrast to the longitudinal mode, the transverse mode is much more damped. However, the dampening factor changes significantly with temperature, also in contrast to the longitudinal case, and at 250 K becomes relatively small. Beyond 2 $Å^{-1}$ the peak due to the damped resonance starts to disappear so values beyond 3 $Å^{-1}$ are not shown.

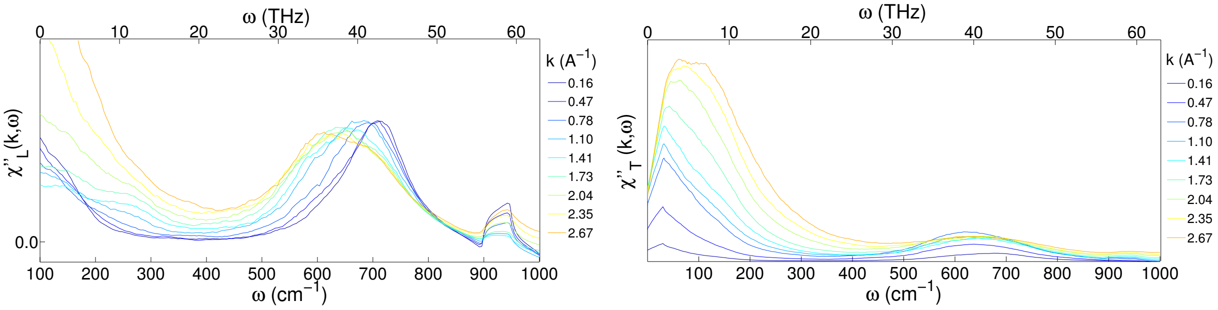

Supplementary Figure 4: **Longitudinal (left) and transverse (right) dielectric susceptibility for a system of 1,000 MeOH molecules.** The longitudinal librational peak at ≈ 700 cm^{-1} clearly disperses with k, while the transverse peak at ≈ 600 cm^{-1} disperses slightly with k. The higher frequency peaks exhibit no dispersion. The static dielectric function $\varepsilon(k, 0)$ has not converged properly in the transverse case, so the magnitude of the peaks is not converged.

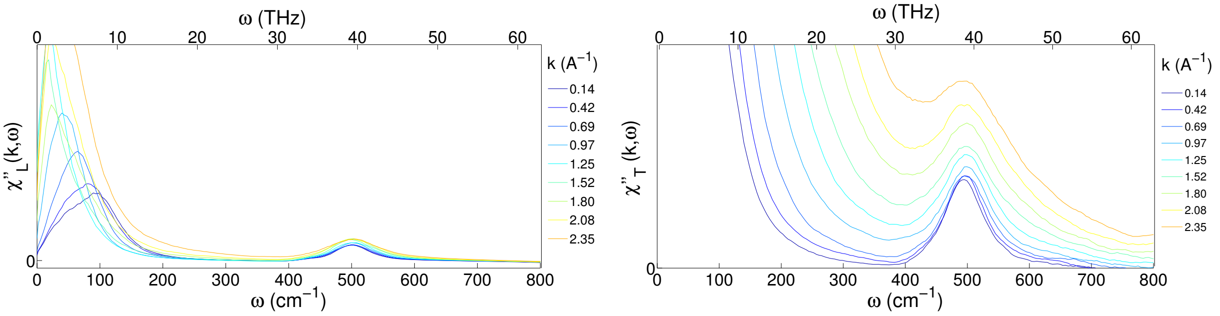

Supplementary Figure 5: **Longitudinal (left) and transverse (right) dielectric susceptibility for a system of 1,000 acetonitrile molecules.** The broad band which peaks at 100 cm^{-1} exhibits dispersion. We hypothesize this dispersion is due entirely to the translational modes, however we cannot say for sure since the librational and translational modes overlap in this region. The peak at ≈ 500 cm^{-1} is due to CCN bending. The static dielectric function $\varepsilon(k, 0)$ has not converged properly, so the magnitude of the transverse peaks is not converged correctly, but the position of the peaks and dispersion can be seen.

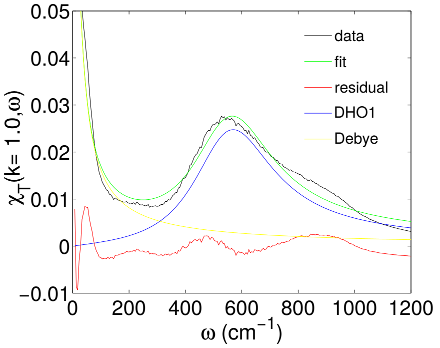

Supplementary Figure 6: **Example fits of the transverse susceptibility of TIP4P/2005f at 300 K.** Fit with a Debye function and one damped harmonic oscillator at $k = .25\text{Å}^{-1}$ and $k = 1.4\text{Å}^{-1}$. The residual show the parts not captured by the fit.

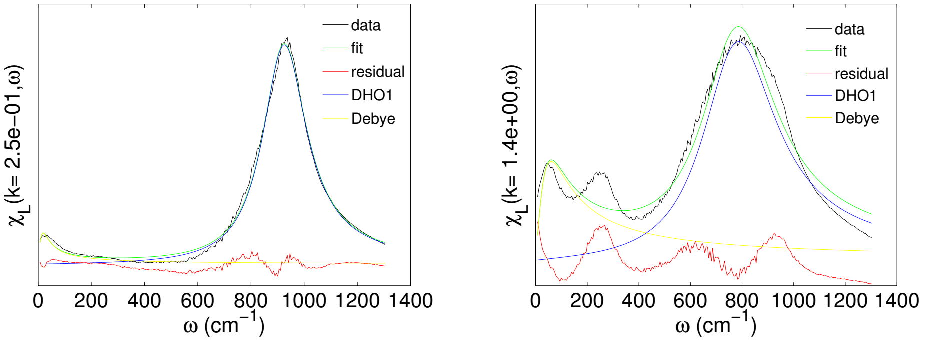

Supplementary Figure 7: **Example fits of the longitudinal susceptibility of TIP4P/2005f at 300K.** Fit with a Debye function and one damped harmonic oscillator at $k = .25\text{Å}^{-1}$ (left) and $k = 1.4\text{Å}^{-1}$ (right). Two peaks appear in the residual - the lower frequency peak is dispersive, having the same dispersion relation as the fitted peak, suggesting that it is actually part of the dispersive peak lineshape that is not captured by our lineshape function. The higher frequency peak in the residual is non-dispersive and is in the same location for both the transverse and longitudinal susceptibility.

type	ω_{L1}	ω_{L2}	ω_{L3}	ref.
	510	—	780	Bolla (1933) [2]
	450	—	780	Walrafen (1962) [3]
	400	—	700	Fukasawa, et. al. (2005)[4]
Raman	430	650	795	Carey, et. al. (1998)[5]
	440	540	770	Castner, et. al. (1995)[6]
	450	550	725	Walrafen (1990)[7]
	424	550	725	Walrafen (1986)[8]
	439	538	717	Walrafen (1967)[9]
infrared	380	665	—	Zelsmann (1995) [10]
dielectric	420	620	—	Fukasawa, et. al. (2005)[4]

Supplementary Table 1: Experimental peaks in Raman, dielectric, and IR spectra. This table shows the correspondence between 3 peak Raman fits and 2 peak dielectric/IR fits to the librational region at 298 K.

Supplementary Note 1

There are several different ways to decompose a spectra into contributions from molecules separated by distance R:

Kirkwood dipole-sphere method

This is the method we choose, which is a modification of the sphere-sphere method (see below). We start with the time-correlation function of interest :

$$\phi(t) = \left\langle \sum_i \boldsymbol{\mu}_i(0) \cdot \sum_j \boldsymbol{\mu}_j(t) \right\rangle \tag{1}$$

Here $\boldsymbol{\mu}$ can be replaced with any dynamical variable of interest, for instance $\boldsymbol{p}^T(\boldsymbol{k}, t)$ or $\boldsymbol{j}(t)$. We omit the k dependence for simplicity.

The most straightforward way is to limit the molecules around each molecule i to those in a sphere of radius R:

$$\phi(t, R) = \left\langle \sum_i \boldsymbol{\mu}_i(0) \cdot \sum_{j \in R_i} \boldsymbol{\mu}_j(t) \right\rangle \tag{2}$$

This is similar to the method employed by Bopp & Kornyshev. During the the course of a simulation molecules enter and leave each sphere, which creates noise, requiring longer averaging times. This can be improved by utilizing a smooth cutoff function:

$$\phi(t, R) = \left\langle \sum_i \boldsymbol{\mu}_i(0) \cdot \sum_j P_{ij}(t) \boldsymbol{\mu}_j(t) \right\rangle \tag{3}$$

where

$$P_{ij} = \frac{1}{1 + e^{(R_{ij} - R)/D}} \tag{4}$$

Here D is a sharpness parameter determining the relative sharpness of the cutoff. We choose not to use smoothing however, finding it to be unnecessary. The result is a spectra $\chi(\boldsymbol{k}, \omega, R)$ showing contributions from molecules up to radius R. The resulting function exhibits the expected $R \to 0$ limit, yielding only the self contribution. In the $R \to \infty$ limit, the original full response function is recovered. This function can then be numerically differentiated to show the contributions from shells of thickness ΔR centered at distance R.

Sphere-sphere method

Another method discussed by Heyden, et. al. (2010) is to study the *autocorrelation* of the total dipole moment of a sphere of radius R centered around a reference molecule, and then average over each molecule in the system.[11]

$$\phi^P(t, R) = \sum_i \left\langle \boldsymbol{\mu}_i^P(0) \cdot \boldsymbol{\mu}_i^P(t) \right\rangle \tag{5}$$

where

$$\boldsymbol{\mu}_i^P(t) = \mathcal{N}_i(t) \sum_{j \in R_i} P_{ij}(t) \boldsymbol{\mu}_j(t) \tag{6}$$

Heyden, et. al. recommend the normalization factor $\mathcal{N}_i(t) = (1 + \sum_{j \in R_i} P_{ij}^2)^{-1/2}$ to normalize for number of molecules in each sphere. This normalization factor is chosen so that in the bulk limit ($R \to \infty$) the original full response function is obtained (in that limit $\mathcal{N}_i = 1/\sqrt{N_{\text{mol}}}$). In the limit $R \to 0$ only the self-term contributes. Results from this method must be interpreted with a bit of care since the calculation includes all cross-correlations between molecules within the sphere centered around the reference molecule. We found that this method is more sensitive to intermolecular correlations, in particular the H-bond stretching at ≈ 250 cm^{-1} (not shown). Altogether though we found the results from this method are complementary with our results from the dipole-sphere method.

Spatial grid method

To achieve higher resolution, Heyden, et. al. also introduce a spatial grid method.[11] The method works by bining the molecular dipoles into grid cells. To reduce noise caused by moleules moving in and out of bins the binning is Gaussian, meaning the dipoles are smeared with a Gaussian function. Unlike the other methods the spatial grid method does not yield the self part as $R \to 0$ so this limit requires special interpretation.

Supplementary Note 2

Bopp & Kornyshev show that to get accurate results in k space it is important to use the polarization vectors for each molecule rather than just the dipole moment. To calculate the polarization vector we use the method of Raineri & Friedman.[12] We utilize the defining relation for the polarization:

$$\nabla \cdot \boldsymbol{P}(\boldsymbol{r}, t) = -\rho(\boldsymbol{r}, t) \tag{7}$$

When transformed into Fourier space this becomes:

$$i\boldsymbol{k} \cdot \boldsymbol{P}(\boldsymbol{r}, t) = -\rho(\boldsymbol{k}, t) \tag{8}$$

We introduce polarization vectors for each molecule $\boldsymbol{p}_i(\boldsymbol{k})$ so that we have

$$\boldsymbol{P}(\boldsymbol{k}) = \sum_i \boldsymbol{p}_i^{N_{\rm mol}}(\boldsymbol{k}) e^{-i\boldsymbol{k}\cdot\boldsymbol{r}_i} \tag{9}$$

where

$$i\boldsymbol{k} \cdot \boldsymbol{p}_i(\boldsymbol{k}) = -\sum_\alpha q_\alpha e^{-i\boldsymbol{k}\cdot\boldsymbol{r}_{\alpha i}} \tag{10}$$

The molecules are indexed by i and the atoms on each molecule are indexed by α. $\boldsymbol{r}_{i\alpha} = \boldsymbol{r}_i(t) - \boldsymbol{r}_\alpha(t)$ is the distance from each atomic site to the center of mass of molecule i. Following Raineri & Friedman, we use the identity

$$e^x = 1 + x \int_0^1 ds e^{xs} \tag{11}$$

and taking into account the charge neutrality of each molecule we obtain

$$\boldsymbol{p}_i(\boldsymbol{k}) = -\sum_\alpha q_\alpha \boldsymbol{r}_{\alpha i} \int_0^1 ds e^{-i\boldsymbol{k}\cdot\boldsymbol{r}_{\alpha i} s} \tag{12}$$

$$\boldsymbol{p}_i(\boldsymbol{k}) = \sum_\alpha \frac{q_\alpha \boldsymbol{r}_{\alpha i}}{i\boldsymbol{k}\cdot\boldsymbol{r}_{\alpha i}} \left(e^{i\boldsymbol{k}\cdot\boldsymbol{r}_{\alpha i}} - 1\right) \tag{13}$$

The transverse part is then calculated as $\boldsymbol{P}_T = \hat{\boldsymbol{k}} \times \boldsymbol{P}$, while the longitudinal component is $\boldsymbol{P}_L = \hat{\boldsymbol{k}} \cdot \boldsymbol{P}$. The longitudinal component can also be calculated more directly from:

$$\hat{\boldsymbol{k}} \cdot \boldsymbol{P} = \frac{i\rho(\boldsymbol{k}, t)}{k} = P_L \tag{14}$$

This yields the following Kubo formula for the longitudinal part of the response:

$$\chi_L(\boldsymbol{k}, \omega) = \frac{\beta}{\epsilon_0 k^2} \int_0^\infty dt \frac{d}{dt} \langle \rho(\boldsymbol{k}, t) \rho^*(\boldsymbol{k}, 0) \rangle e^{i\omega t} \tag{15}$$

For a system composed of point charges, the charge density is :

$$\rho(\boldsymbol{r}, t) = \frac{1}{V} \sum_i \sum_\alpha q_{i\alpha} \delta(\boldsymbol{r} - \boldsymbol{r}_i(t) - \boldsymbol{r}_{i\alpha}(t)) \tag{16}$$

Again, the index i runs over the molecules while α runs over the atomic sites on each molecule. The charge density in k-space becomes:

$$\rho(\boldsymbol{k}, t) = \frac{1}{V} \sum_i \sum_\alpha q_\alpha e^{-i\boldsymbol{k}\cdot(\boldsymbol{r}_i(t) + \boldsymbol{r}_{i\alpha}(t))} \tag{17}$$

Note that this can be Taylor expanded as:

$$\begin{aligned} \frac{\rho(\boldsymbol{k}, t)}{k} &= \frac{1}{k} \sum_i \sum_\alpha q_\alpha \sum_n \frac{(-i\boldsymbol{k}\cdot\boldsymbol{r}_{i\alpha}(t))^n}{n!} \\ &= \boldsymbol{M}(\boldsymbol{k}, t) + \boldsymbol{\mathcal{Q}}(\boldsymbol{k}, t) + \boldsymbol{\mathcal{O}}(\boldsymbol{k}, t) + \cdots \end{aligned} \tag{18}$$

Here $\boldsymbol{M}(\boldsymbol{k},t)$, $\boldsymbol{\mathcal{Q}}(\boldsymbol{k},t)$, $\boldsymbol{\mathcal{O}}(\boldsymbol{k},t)$ are contributions due to the molecular dipoles, quadrupoles and octupoles. In the limit $k \rightarrow 0$ it from supplementary equation 15 it can be seen that only the dipole term contributes to the susceptibility. In the $k \rightarrow 0$ limit one obtains

$$\chi_L(k,\omega) \approx \frac{\beta}{3\epsilon_0 V} \int_0^\infty dt \frac{d}{dt} \langle \boldsymbol{M}_L(\boldsymbol{k},t) \cdot \boldsymbol{M}_L^*(\boldsymbol{k},0) \rangle e^{i\omega t} \tag{19}$$

with

$$\boldsymbol{M}_L(\boldsymbol{k},t) = \sum_{i=1}^{N_{\text{mol}}} \hat{\boldsymbol{k}} \cdot \boldsymbol{\mu}_i(t) e^{i\boldsymbol{k}\cdot\boldsymbol{r}_i(t)} \tag{20}$$

This type of expression has been used previously as an approximate expression at small k.[13] However, Bopp & Kornyshev show quite convincingly that for water the higher order multipole terms are very important, even at the smallest k available in computer simulation.[14] Neglect of the higher order terms leads to severe consequences at large k, and one will not recover the physical limit $\lim_{k\rightarrow\infty} \chi_{L/T}(k) = 1$ unless higher order terms are included.

Supplementary references

[1] Buchner, R., Barthel, J. & Stauber, J. The dielectric relaxation of water between 0c and 35c. *Chem. Phys. Lett.* **306**, 57 (1999).

[2] Bolla, G. Su alcune nuove bande Raman dellacqua. *Il Nuovo Cimento* **10**, 101–107 (1933).

[3] Walrafen, G. E. Raman spectral studies of the effects of electrolytes on water. *J. Chem. Phys.* **36**, 1035–1042 (1962).

[4] Fukasawa, T. *et al.* Relation between dielectric and low-frequency Raman spectra of hydrogen-bond liquids. *Phys. Rev. Lett.* **95**, 197802 (2005).

[5] Carey, D. M. & Korenowski, G. M. Measurement of the Raman spectrum of liquid water. *J. Chem. Phys.* **108**, 2669–2675 (1998).

[6] Castner, E. W., Chang, Y. J., Chu, Y. C. & Walrafen, G. E. The intermolecular dynamics of liquid water. *J. Chem. Phys.* **102**, 653–659 (1995).

[7] Walrafen, G. E. Raman spectrum of water: transverse and longitudinal acoustic modes below ≈ 300 cm^{-1} and optic modes above ≈ 300 cm^{-1}. *J. Phys. Chem.* **94**, 2237–2239 (1990).

[8] Walrafen, G. E., Fisher, M. R., Hokmabadi, M. S. & Yang, W. Temperature dependence of the low and highfrequency Raman scattering from liquid water. *J. Chem. Phys.* **85**, 6970–6982 (1986).

[9] Walrafen, G. E. Raman spectral studies of the effects of temperature on water structure. *J. Phys. Chem.* **47**, 114–126 (1967).

[10] Zelsmann, H. R. Temperature dependence of the optical constants for liquid H_2O and D_2O in the far IR region. *J. Mol. Str.* **350**, 95–114 (1995).

[11] Heyden, M. *et al.* Dissecting the THz spectrum of liquid water from first principles via correlations in time and space. *Proc. Natl. Acad. Sci. USA* **107**, 12068–12073 (2010).

[12] Raineri, F. O. & Friedman, H. L. Static transverse dielectric function of model molecular fluids. *J. Chem. Phys.* **98**, 8910–8918 (1993).

[13] Bertolini, D. & Tani, A. The frequency and wavelength dependent dielectric permittivity of water. *Mol. Phys.* **75**, 1065–1088 (1992).

[14] Bopp, P. A., Kornyshev, A. A. & Sutmann, G. Frequency and wave-vector dependent dielectric function of water: Collective modes and relaxation spectra. *J. Chem. Phys.* **109**, 1939 (1998).
